# Network-based mapping and neurotransmitter architecture of gray matter correlates of neuroticism

**DOI:** 10.3389/fnsys.2025.1713434

**Published:** 2026-01-08

**Authors:** Shu Wang, Hu-Cheng Yang, Hai-Hua Sun, Feng-Mei Zhang, Zhen-Yu Dai, Ping-Lei Pan, Si-Yu Gu

**Affiliations:** 1 Department of Radiology, Affiliated Hospital 6 of Nantong University, Yancheng Third People’s Hospital, Yancheng, China; 2 Department of Radiology, Binhai Maternal and Child Health Hospital, Yancheng, China; 3 Department of Geriatrics, Affiliated Hospital 6 of Nantong University, Yancheng Third People’s Hospital, Yancheng, China; 4 Department of Neurology, Affiliated Hospital 6 of Nantong University, Yancheng Third People’s Hospital, Yancheng, China

**Keywords:** default mode network, frontoparietal network, functional connectivity network mapping, gray matter, neuroticism, ventral attention network

## Abstract

**Objectives:**

Although neuroticism is a major risk factor for adverse health outcomes, its neural basis is obscured by inconsistent findings from studies of regional gray matter volume (GMV) correlates. This study sought to identify convergent functional brain networks underlying these heterogeneous GMV correlates using functional connectivity network mapping (FCNM), and to explore their neurochemical basis.

**Methods:**

We systematically identified 10 voxel-based morphometry (VBM) studies (*N* = 1,595) reporting neuroticism-associated GMV coordinates. Using resting-state fMRI data from 1,093 healthy Human Connectome Project participants, FCNM was applied to map functional connectivity patterns associated with these coordinates. Overlap with canonical networks was assessed. The Juspace toolbox explored spatial relationships between identified networks and major neurotransmitter receptor distributions.

**Results:**

Despite spatial heterogeneity, neuroticism-related GMV changes consistently mapped onto three principal functional networks: the default mode network (DMN), frontoparietal network (FPN), and ventral attention network (VAN). These mappings were robust across varied analytical parameters. Moreover, the implicated networks demonstrated significant spatial correlation with the distributions of 5-hydroxytryptamine receptor 2A (5-HT2A), cannabinoid receptor type 1 (CB1), and metabotropic glutamate receptor 5 (mGluR5).

**Conclusion:**

Despite regional variability, GMV correlates of neuroticism converge on common large-scale brain networks involved in self-referential processing, cognitive control, and salience processing. Their significant spatial coupling with 5-HT2A, CB1, and mGluR5 receptor distributions suggests serotonergic, endocannabinoid, and glutamatergic modulatory mechanisms contributing to network-level alterations. This cross-modal and network-based approach provides a unified framework for understanding the biological substrates of neuroticism, reconciling prior inconsistencies, and identifying key targets for prevention or biomarker development.

## Introduction

Neuroticism, a fundamental dimension of human personality, represents a stable, heritable tendency to experience frequent and intense negative emotions such as anxiety, sadness, anger, and worry (Barlow et al., 2014; Ormel et al., 2013a). This trait extends beyond typical emotional responses, acting as a significant vulnerability factor for a broad array of common mental disorders, including depression and anxiety (Barlow et al., 2014, 2021; Lahey, 2009; Ormel et al., 2013a). Furthermore, compelling evidence links higher neuroticism scores to increased susceptibility to various physical health problems and even reduced longevity (Lahey, 2009; Mader et al., 2023; Mahmood et al., 2023). Given its profound public health significance and its pervasive impact on quality of life, understanding the biological and psychological basis of neuroticism is crucial for developing effective strategies to prevent or mitigate its wide-ranging adverse outcomes (Lin et al., 2023; Ormel et al., 2013a).

Over the past two decades, neuroimaging techniques, particularly magnetic resonance imaging (MRI), have advanced our ability to investigate the structural correlates of personality traits. Voxel-based morphometry (VBM) to assess regional gray matter volume (GMV), an established metric of brain structure, has been extensively employed to explore the neural basis of neuroticism (Ashburner and Friston, 2000; Liu et al., 2021). Numerous studies have examined the relationship between GMV and neuroticism scores, aiming to identify specific brain regions associated with this trait (DeYoung et al., 2010; Du et al., 2015; Kapogiannis et al., 2013; Lin et al., 2022; Liu et al., 2021; Lu et al., 2014; Nostro et al., 2017; Omura et al., 2005; Yang et al., 2017; Zhang et al., 2022). While certain regions, such as the prefrontal cortex, areas responsible for emotional processing, self-regulation, and negative affect, have been repeatedly implicated, and are thought to contribute to the emotional instability and vulnerability seen in individuals high in neuroticism, these investigations have yielded somewhat fragmented and inconsistent findings.

These inconsistencies suggest that a purely regional approach may be insufficient to capture the complexity of neuroticism. It is increasingly recognized that complex psychological traits like neuroticism are not confined to isolated brain regions, but likely arise from the integrated function of distributed brain networks. Recent advances in network neuroscience offer an opportunity to reconceptualize heterogeneous neuroimaging findings within a unified framework (Darby et al., 2019). Network mapping approaches have successfully identified common brain networks underlying various neuropsychiatric conditions and symptoms despite heterogeneous regional findings (Burke et al., 2020; Stubbs et al., 2023; Wang Y. et al., 2024; Xu et al., 2024). Applying this network perspective to neuroticism could potentially resolve prior inconsistencies regarding GMV variations and offer a more biologically plausible model of its neural substrates. Functional connectivity network mapping (FCNM) offers a powerful and increasingly validated methodology for this purpose (Cheng et al., 2025; Mo et al., 2024; Zhang et al., 2024). By leveraging large-scale normative human connectome data, typically from resources like the Human Connectome Project (HCP) (Glasser et al., 2016), FCNM allows spatially disparate structural findings (like GMV) to be mapped onto common underlying functional connectivity (FC) networks.

Beyond identifying the relevant brain networks, understanding their underlying molecular mechanisms is critical for a comprehensive picture of neuroticism. Dysregulation of neurotransmitter systems, such as serotonin, has long been associated with mood, anxiety, and personality (Hettema et al., 2006; Mandelli et al., 2012; Takano et al., 2007; Tuominen et al., 2017; Zmorzyński et al., 2021). Recent advancements, such as the JuSpace toolbox, provide a novel technique for cross-modal spatial correlation between MRI-derived network maps and atlas-based distributions of various positron emission tomography (PET) and single-photon computed emission tomography (SPECT) radioligands (Dukart et al., 2020). This allows for the characterization of the neurotransmitter receptor and transporter landscape of specific brain networks (Lan et al., 2025; Li et al., 2024; Sun et al., 2024). While JuSpace has been applied to uncover neurotransmitter correlates of brain alterations in various neurological and psychiatric disorders (Dugré and Potvin, 2022; Lan et al., 2025; Morais et al., 2025; Premi et al., 2023; Tang et al., 2025), its application to elucidate the neurotransmitter architecture of brain networks associated with personality traits like neuroticism remains unexplored.

Therefore, this study aimed to integrate these advanced neuroimaging and analytical approaches. First, we utilized FCNM to synthesize existing literature on GMV correlates of neuroticism, aiming to identify a common brain network. Second, we employed the JuSpace toolbox to investigate the neurotransmitter receptor and transporter architecture of this identified neuroticism-related network. By combining network mapping with neurotransmitter profiling, we sought to provide a more comprehensive understanding of the brain network and neurochemical basis of neuroticism, potentially bridging the gap between macro-level brain alterations and their micro-level molecular underpinnings. This integrated approach may offer novel insights into the biological mechanisms contributing to neuroticism and inform future targeted interventions.

## Materials and methods

### Literature search and selection

Following the PRISMA guidelines, we conducted a comprehensive and systematic search of the PubMed, Embase and Web of Science databases to identify VBM studies on GM correlates of neuroticism, published prior to 25 December 2024. The search included keywords such as “voxel-based morphometry” OR “VBM” OR “gray matter” OR “gray matter” AND “neuroticism” OR “emotional stability.” We also conducted a manual examination of the bibliographies from pertinent review articles and meta-analyses to capture any studies that may have been missed through the primary search strategy. A flow diagram of the study selection process is shown in Supplementary Figure 1. All studies were included according to the following criteria: (a) published in an English-language peer-reviewed journal as an original article; (b) examined healthy subjects; (c) focused on neuroticism (emotional stability) as the primary research variable; (d) used whole-brain voxel-based analysis; (e) reported regional GM correlates of neuroticism; (f) reported results in Talairach or Montreal Neurological Institute (MNI) space. Exclusion criteria were as follows: (a) non-original studies (e.g., review, meta-analysis, meeting abstract); (b) no coordinate system reported; (c) only reported regions of interest (ROIs) results; (d) all reported coordinates outside the GM mask; and (e) studies involving animal experiments. To avoid data duplication from overlapping patient samples across different publications, only the study reporting the largest sample size and most comprehensive information was retained for analysis. Two investigators (S. W. and H. C. Y.) independently performed literature search and selection and data extraction. Any discrepancies were discussed with another investigator (P. L. P.) until they were resolved.

### Functional MRI data acquisition and preprocessing

This study utilized resting-state fMRI data from the HCP 1200 Subjects Release (S1200), which comprises imaging data from healthy young adults aged 22–37 years. Our final analysis included 1,093 healthy participants [594 female; mean (SD) age = 28.78 (3.69) years]. Participants were excluded based on criteria such as contraindications to MRI, current psychiatric or neurological disorders, psychotropic medication use within the past 3 months, pregnancy, or a history of head trauma. The demographic details of the HCP dataset are presented in Supplementary Table 1. All coordinates extracted from the previous studies were uniformly converted to the MNI standard space.^1^

Resting-state fMRI data were acquired from the HCP using a 3T Siemens Trio MRI scanner. Specific MRI acquisition parameters for this dataset are detailed in Supplementary Table 2. Participants with images of insufficient quality, including those affected by artifacts or lacking complete brain coverage, were excluded from the analysis.

We carried out preprocessing of the resting-state fMRI data using SPM12 software^2^ and the DPABI toolbox^3^. For each participant, the initial 10 scan volumes were discarded to ensure signal equilibrium and allow for participant adaptation to the scanner noise. The remaining volumes were corrected for the acquisition time delay between slices. Then, realignment was performed to correct for motion between time points. Head motion parameters comprised the estimated translation in each direction and angular rotation on each axis, calculated for every volume. All included participants met the predefined criteria for head motion, with maximum translational and rotational movements not exceeding 2 mm and 2°, respectively. We also calculated framewise displacement, which indexes the volume-to-volume changes in head position. Several nuisance covariates (linear drift, estimated motion parameters based on the Friston-24 model, spike volumes with framewise displacement > 0.5 mm, global signal, white matter signal, and cerebrospinal fluid signal) were regressed out from the data. Because global signal regression can enhance the detection of system-specific correlations and improve the correspondence to anatomical connectivity, we included this step in the preprocessing of resting-state fMRI data. Then, the datasets were bandpass filtered using a frequency range of 0.01–0.1 Hz. In the normalization step we segmented and normalized the transformed structural images to MNI space. Subsequently, each filtered functional volume was spatially normalized to MNI space using the deformation parameters obtained in the previous step and resampled to a 3-mm isotropic voxel grid. Finally, we spatially smoothed all data with a Gaussian kernel of 6 × 6 × 6 mm^3^ full width at half maximum.

### FCNM analysis

We utilized the FCNM approach to map regional GM correlates of neuroticism to dysfunctional networks. First, spheres with a 4-mm radius were centered at each coordinate of a contrast, and these spheres were combined to create a contrast-specific seed mask (henceforth referred to as the contrast seed). Second, a seed-to-whole brain FC map was constructed for each participant using the preprocessed resting-state fMRI data from the HCP. This process included computing Pearson’s correlation coefficients between the seed’s time series and all brain voxels, followed by a Fisher’s Z-transformation to enhance normal distribution. Third, the FC maps from 1,093 subjects were analyzed using a voxel-wise one-sample *t*-test to pinpoint brain regions functionally linked to each seed. We focused solely on positive FC, as the interpretation of negative connectivity remains controversial (Murphy and Fox, 2017; Murphy et al., 2009). Fourth, the resulting group-level *t* maps were thresholded and binarized at *P* < 0.05 corrected for multiple comparisons using the voxel-level false discovery rate method. We opted for voxel-level correction over cluster-level inference to ensure precise spatial localization of the identified FC patterns, irrespective of cluster size. Finally, the binarized FC maps corresponding to all included contrasts were overlaid to form a network probability map. This map was then thresholded at 50% overlap (i.e., regions connected to at least 50% of the contrast seeds), a threshold validated in previous FCNM studies to identify the core and robustly connected network while balancing sensitivity and specificity (Cheng et al., 2025; Peng et al., 2022; Zhang et al., 2024). Moreover, we conducted sensitivity analyses to ensure that our findings were not influenced by random parameter selections. These analyses involved utilizing seed spheres with radii of 1 mm and 7 mm, resulting in remarkably consistent network topographies.

### Association with canonical brain networks

For ease of interpretability, we examined the spatial relationships between the identified neuroticism-related brain dysfunctional networks and 8 well-recognized canonical brain networks. The seven cortical networks were defined as the visual network, somatomotor network, dorsal attention network, ventral attention network (VAN), limbic network, frontoparietal network (FPN), and default mode network (DMN) according to the Yeo et al. (2011) study. The Human Brainnetome Atlas (Fan et al., 2016) was utilized to delineate the subcortical network. We quantified their spatial relationships by calculating the ratio of overlapping voxels between each neuroticism-related dysfunctional network and the respective canonical network to the total number of voxels within that canonical network.

### Neurotransmitter analysis

To elucidate the relationship between the neuroticism mapping networks and chemoarchitectonic organization, we conducted spatial correlation analysis using 30 PET-derived neurotransmitter transporter and receptor maps available in JuSpace version 1.5^4^ (Dukart et al., 2021). The significance of each spatial correlation between the neuroticism network map and the 30 neurotransmitter maps was evaluated through a permutation test consisting of 5,000 iterations. Subsequently, the obtained *p*-values for all 30 correlations were adjusted for multiple comparisons using the false discovery rate (FDR) method, with significance established at *p* < 0.05.

## Results

### Included studies and sample characteristics

Following the systematic literature search and screening process detailed in Supplementary Figure 1, a total of 10 VBM studies (Cremers et al., 2011; DeYoung et al., 2010; Du et al., 2015; Kapogiannis et al., 2013; Lin et al., 2022; Lu et al., 2014; Nostro et al., 2017; Omura et al., 2005; Yang et al., 2017; Zhang et al., 2022) involving 1,595 participants (879 female,716 male) were included for the subsequent network mapping analysis. Detailed sample and imaging characteristics of the included studies are summarized in Table 1.

**TABLE 1:** Sample and imaging characteristics of the studies included neuroticism analysis.

References	Sample size	Ratio F/M	Mean age (SD)	Scales	Scanner/FWHM (mm)	Nuisance covariate	Statistical analysis/*p*-value
Cremers et al., 2011	65	42/23	40.5 (9.7)	NEO-FFI	3.0 T/8	Age, scan center, TGMV	GLM/*p* < 0.05, FWE corr & *p* < 0.001, uncorr
Du et al., 2015	298	158/140	19.94 (1.26)	NEO-PI-R	3.0 T/–	Gender, age, TGMV	Multiple regression/*p* < 0.05, non-stationary cluster corr
DeYoung et al., 2010	116	58/58	22.9 (5.5)	NEO-PI-R	3.0 T/8	Gender, age, TGMV	GLM/*p* < 0.05, cluster-size corr (Monte Carlo simulation)
Kapogiannis et al., 2013	87	42/45	72 (7.7)	NEO-PI-R	1.5 T/12	TIV, years of education	GLM/*p* < 0.05, FWE corr
Lu et al., 2014	71	37/34	22.35 (1.5)	EPQ-RSC	3.0 T/8	Gender, age, TIV	GLM/*p* < 0.05, AlphaSim corr
Lin et al., 2022	298	158/140	19.94 (1.28)	NEO-PI-R	3.0 T/–	Gender, age, TIV	Multiple regression/*p* < 0.05, non-stationary cluster corr
Omura et al., 2005	41	22/19	23.8 (5.4)	NEO-PI-R	3.0 T/12	Gender, age	GLM/*p* < 0.001, uncorr
Nostro et al., 2017	364	182/182	29.1 (3.45)	NEO-FFI	3.0 T/8	Gender, age, TIV	GLM/*p* < 0.05, FWE corr
Yang et al., 2017	356	200/156	20.00 (1.32)	NEO-PI-R	3.0 T/10	Gender, age, TGMV, intelligence, family income, education years of parents	Multiple regression/*p* < 0.05, non-stationary cluster corr
Zhang et al., 2022	153	112/41	20.20 (1.85)	NEO-FFI	3.0T/8	Gender, age, TGMV	GLM/*p* < 0.05, GRF corr

corr, correction; EPQ-RSC, Eysenck Personality Questionnaire-Revised Short Scale for Chinese; F, female; FWE, family-wise error; FWHM, full width at half maximum; GLM, general linear model; GRF, Gaussian random field; M, male; NEO-FFI, NEO Five Factor Inventory; NEO-PI-R, Revised NEO Personality Inventory; TGMV, total gray matter volume; TIV, total intracranial volumes; uncorr, uncorrection.

### Convergent FC associated with heterogenous GM correlates of neuroticism

**FIGURE 1:**
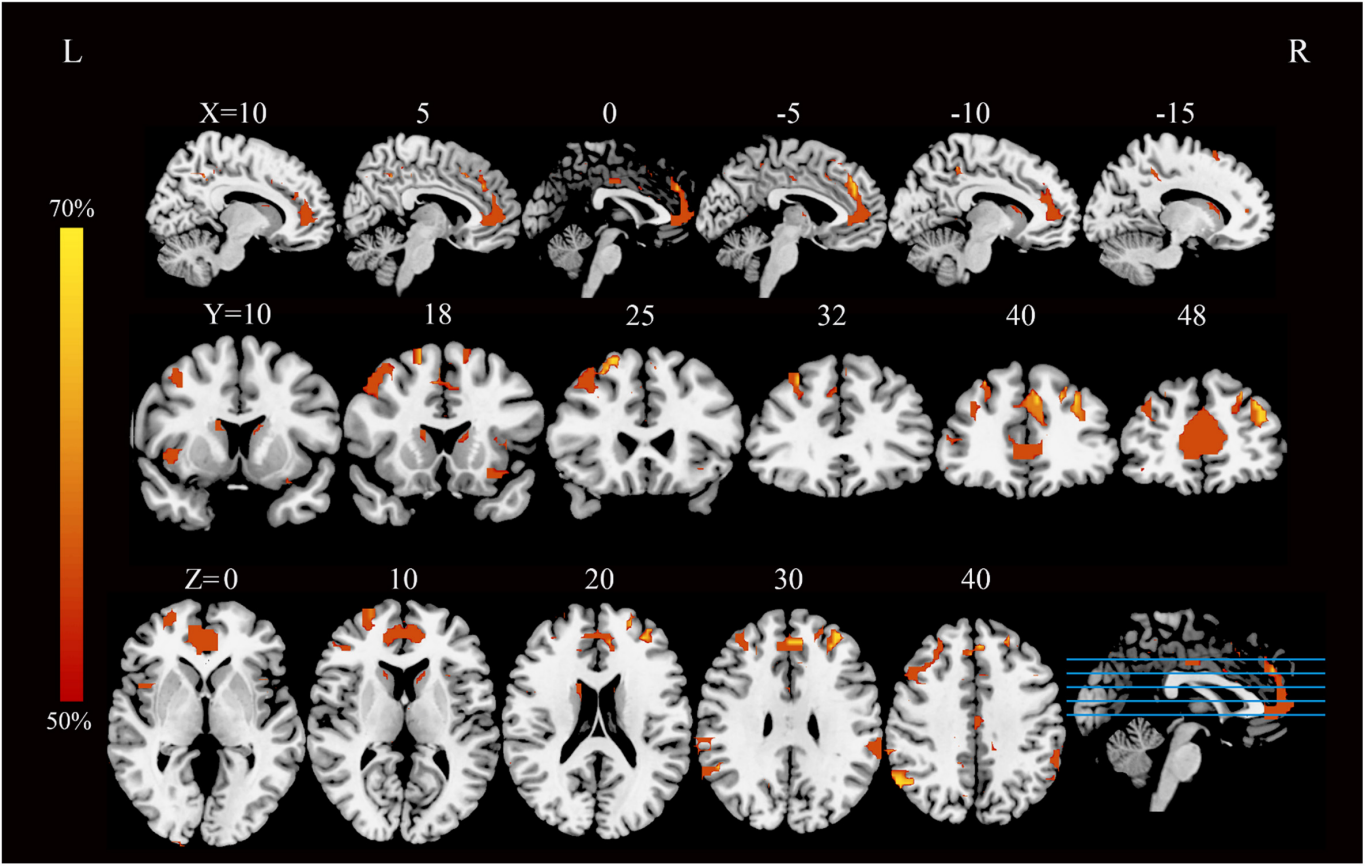
Neuroticism-associated FC overlap map (4-mm sphere radius). This FC map illustrates brain regions functionally connected to more than 50% of the contrast seeds derived from neuroticism-related gray matter coordinates, based on a 4-mm sphere radius analysis. FC, functional connectivity; L, left; R, right.

Application of the FCNM approach, integrating coordinates of reported GM correlates of neuroticism from the 10 VBM studies with the normative HCP connectome, revealed convergent aberrant FC associated with heterogenous GM correlates of neuroticism. These aberrant FC patterns involved widely distributed brain regions, primarily including the bilateral medial prefrontal cortex (PFC), bilateral anterior PFC, bilateral precuneus/posterior cingulate cortex (PCC), dorsolateral prefrontal cortex (DLPFC), and bilateral temporoparietal junction (TPJ) (Figure 1).

### Association with canonical brain networks

**FIGURE 2:**
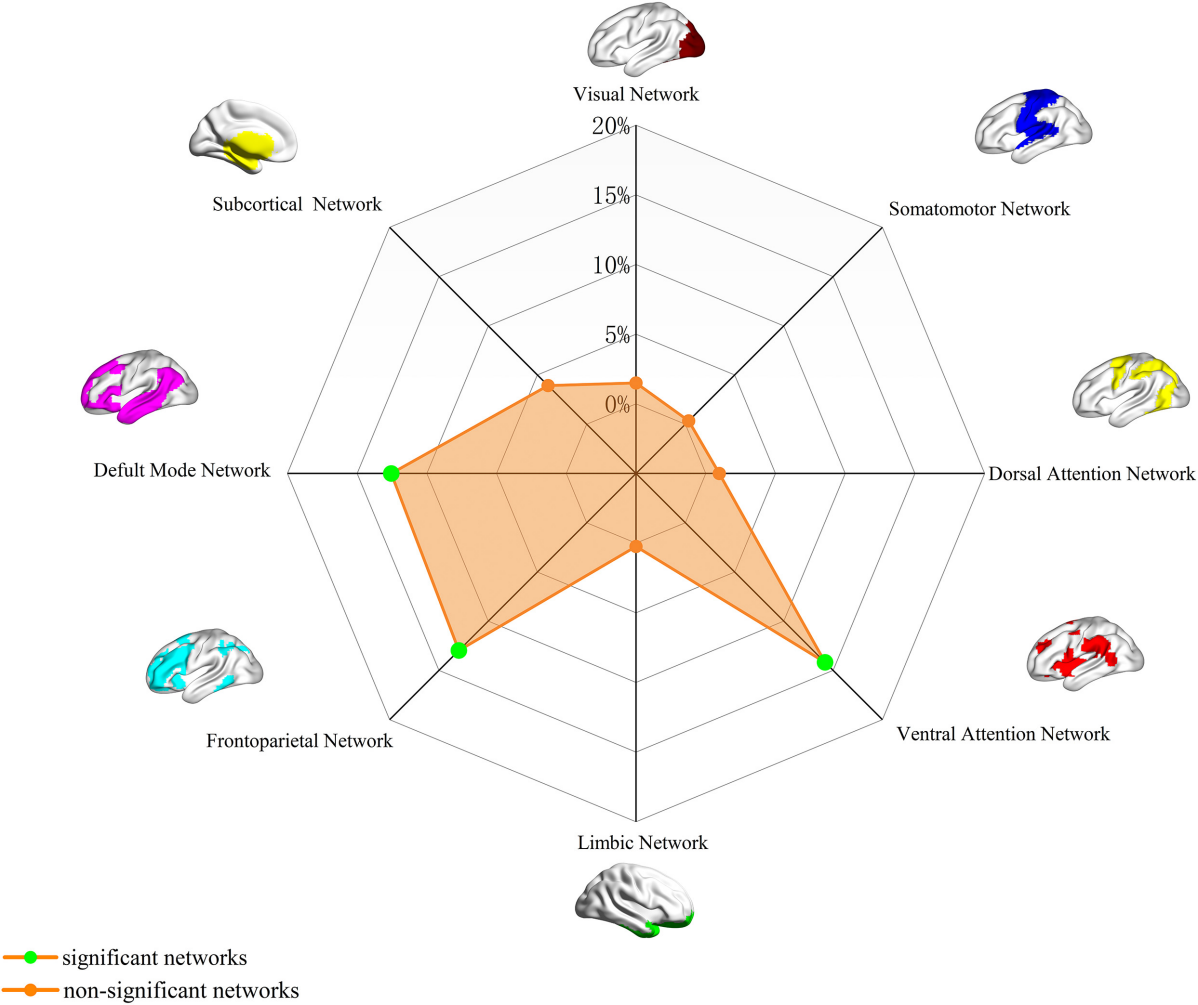
Neuroticism-associated FC overlap map based on 4-mm radius sphere in association with canonical brain networks. Polar plots illustrate the proportion of overlapping voxels between each neuroticism FC map and a canonical network to all voxels within the corresponding canonical network. The blue dot represents brain functional networks, defined as significant networks, exhibiting ≥10% overlap with canonical networks, whereas the orange dot represents non-significant networks with <10% overlap. FC, functional connectivity.

Analysis of the spatial overlap between the neuroticism-related aberrant FC map and established canonical brain networks indicated preferential involvement of specific systems. The network showed the highest overlap with the VAN, largely corresponding to the bilateral TPJ (overlap proportion: 14.16%). Significant overlap was also observed with the FPN, primarily involving the bilateral anterior PFC and bilateral DLPFC (overlap proportion: 12.96%), and the DMN, encompassing the bilateral medial PFC and bilateral precuneus/PCC (overlap proportion: 12.55%) (Figure 2). Overlap proportions with the remaining canonical networks (visual, somatomotor, dorsal attention, limbic, subcortical) were all below 10%.

### Robustness analyses

Functional connectivity network mapping analyses repeated using seed spheres with 1- and 7-mm radii yielded topographically similar network patterns to those obtained using the standard 4-mm radius sphere (Supplementary Figures 2, 3, respectively). Furthermore, the pattern of canonical network involvement remained consistent when replicating the FCNM procedure with spheres of 1- and 7-mm radii (Supplementary Figures 4, 5, respectively).

### Neurotransmitters associated with brain networks correlates of neuroticism

**FIGURE 3:**
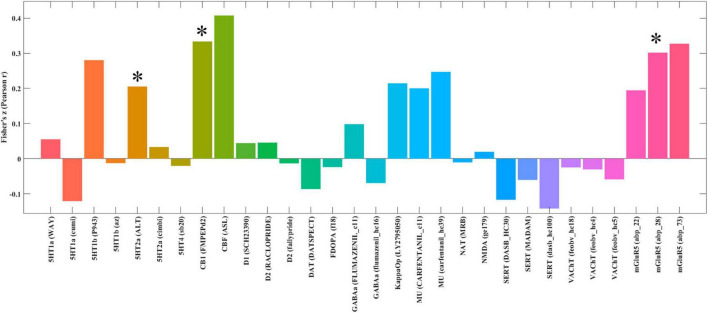
Spatial correlation between brain networks associated with neuroticism and neurotransmitter system maps. The figure displays brain regions where the statistical map of brain networks associated with neuroticism shows significant spatial correlation with predefined maps of neurotransmitter distributions. *Indicates significant correlation at *p* < 0.05, corrected for multiple comparisons using the False Discovery Rate (FDR) method. 5HT1A, 5-Hydroxytryptamine Receptor 1A; 5HT1B, 5-Hydroxytryptamine Receptor 1B; 5HT2A, 5-Hydroxytryptamine Receptor 2A; 5HT4, 5-Hydroxytryptamine Receptor 4; CB1, Cannabinoid Receptor type 1; D1, Dopamine Receptor D1; D2, Dopamine Receptor D2; DAT, Dopamine Transporter; FDOPA, Fluorodopa; GABAa, Gamma-Aminobutyric Acid type A receptor; KappaOp, Kappa Opioid Receptor; MU, Mu Opioid Receptor; NAT, Noradrenaline Transporter; NMDA, N-Methyl-D-Aspartate Receptor; SERT, Serotonin Transporter; VAChT, Vesicular Acetylcholine Transporter; mGluR5, Metabotropic Glutamate Receptor 5.

Spatial correlation analysis revealed that the pattern of brain networks associated with neuroticism was positively correlated with the distribution of the 5-hydroxytryptamine receptor 2A (5HT2a) (*r* = 0.205, *p* = 0.023), cannabinoid receptor type-1 (CB1) (*r* = 0.333, *p* = 0.038), and metabotropic glutamate receptor 5 (mGluR5) (*r* = 0.327, *p* = 0.010) (Figure 3).

## Discussion

This study, to our knowledge, represents the first to combine the FCNM approach with large-scale resting-state fMRI data from the HCP to map heterogeneous GM correlates of neuroticism onto common brain networks, and to subsequently explore their underlying neurotransmitter receptor architecture. Our analysis drew on 10 VBM studies, encompassing 1,595 participants, and pinpointed critical brain areas tied to correlates of neuroticism, such as the bilateral medial PFC, bilateral precuneus/PCC, bilateral anterior PFC, bilateral DLPFC, and bilateral TPJ. These regions were assigned to three well-established canonical networks: the DMN, (encompassing the bilateral medial PFC and bilateral precuneus/PCC), the FPN (including the bilateral anterior PFC and the bilateral DLPFC), and the VAN (involving the bilateral TPJ). Crucially, further analysis using the JuSpace toolbox demonstrated significant spatial coupling between these neuroticism-implicated networks and the distributions of key neurotransmitter receptors, specifically the 5-HT2A, CB1, and mGluR5. This integrated, cross-modal approach, robust across varying analytical parameters (1-, 4-, and 7-mm), highlights not only the reliability of these identified neural systems but also their distinct neurochemical signatures in the context of neuroticism, offering a more comprehensive understanding of its biological underpinnings.

Consistent with their roles in the established canonical brain networks, the DMN is a collection of interconnected brain regions such as the medial PFC, PCC, angular gyrus, and precuneus (Menon, 2023). The DMN, especially the nodes of the medial PFC and precuneus/PCC identified in our study, plays a crucial role in cognitive and emotional processing, particularly processes involved in self-referential thinking, autobiographical memory, and emotional regulation (Menon, 2023; Sambuco, 2024; Satpute and Lindquist, 2019). Our study identified that heterogeneous GM correlates of neuroticism can be convergently mapped onto the DMN. It has been proposed that neuroticism stems from a predisposition toward negative self-generated thought, driven by individual differences in DMN activity (Perkins et al., 2015). In recent years, growing evidence has emerged linking the DMN to neuroticism, showing correlates of GM structure, biochemical integrity, functional activity, and FC within the DMN that may explain the tendency for self-referential thinking, rumination, worry, and emotional dysregulation commonly observed in highly neurotic individuals (Dong et al., 2020; Eisenberger et al., 2005; Gentili et al., 2017; Lee et al., 2024; Liu et al., 2021; Ryman et al., 2011; Servaas et al., 2013, 2014; Song et al., 2015; Wei et al., 2016; Zhi et al., 2024). Additionally, neuroticism is not only related to the DMN but also involves its interaction with other brain networks; this interaction is crucial for understanding how neuroticism manifests in specific psychological processes, influencing individuals’ emotional and cognitive states (Cai et al., 2020; Lee et al., 2024; Wang and Chen, 2024). Importantly, evidence from the connectome-based predictive modeling of resting-state fMRI data from the HCP has shown that the DMN is a key component of the brain network that predicts individual differences in neuroticism (Cai et al., 2020). Furthermore, genetic predisposition to neuroticism is linked to both brain structural and functional regions associated with the DMN (Opel et al., 2020; Servaas et al., 2017) that may contribute to the development of neurotic personality traits, and potentially increase vulnerability to mental health disorders like MDD (Opel et al., 2020). Taken together, these findings highlight the critical role of the DMN, particularly the medial PFC and precuneus/PCC, in the neural underpinnings of neuroticism. Given the involvement of the DMN in neuroticism, interventions such as emotion regulation therapy, mindfulness-based therapies, cognitive behavioral therapy, and neuromodulation techniques like rTMS, which have demonstrated the capacity to modulate DMN activity and reduce rumination and enhance emotional regulation, offer targeted therapeutic strategies for addressing neuroticism-related traits (Scult et al., 2019).

The FPN, also known as the central executive network, is crucial for cognitive control, working memory, goal-directed behavior, and emotion regulation (Gao et al., 2024; Otstavnov et al., 2024). It connects regions in the frontal cortex, such as the anterior PFC and DLPFC, with areas in the parietal cortex, facilitating the integration of cognitive and emotional processes. Our findings contribute to the growing body of literature implicating the FPN in the neurobiological underpinnings of neuroticism. Research indicates that neuroticism is associated with FC patterns within the FPN, which may reflect an imbalance in cognitive and emotional processing in individuals with neuroticism (Dima et al., 2015; Ormel et al., 2013b; Servaas et al., 2017; Xia et al., 2024). The findings of Servaas et al. (2017) underscore that neuroticism was associated with reduced functional integration and connectivity in the FPN, particularly in 5-HTTLPR S-carriers. Ueda et al. (2018) further provided evidence that neuroticism is associated with reduced structural connectivity in DMN and FPN hubs, which reflects neuroticism as a systems-level disorder characterized by structural disconnection in control-affect pathways. FPN dysfunction may contribute to cognitive control and emotion regulation problems in individuals with neuroticism, increasing the risk of psychiatric disorders (Ormel et al., 2013b; Xia et al., 2024).

Our findings lend further support to the emerging consensus that the VAN plays a significant role in the neurobiological underpinnings of neuroticism. The VAN, which includes key regions such as the right TPJ, ventrolateral prefrontal cortex, and inferior frontal gyrus, is thought to play a role in bottom-up attention and in processing emotionally salient information (Viviani, 2013). Individuals characterized by high neuroticism may demonstrate a persistent attentional bias toward threatening or self-relevant negative information due to a hyper-responsive or dysregulated VAN (Hu et al., 2024). This model is highly consistent with reports linking aberrant VAN connectivity to rumination and anxiety symptoms, both core facets of the neuroticism construct (Jylhä and Isometsä, 2006; Vidal-Arenas et al., 2022; Wang X. et al., 2024). Indeed, higher levels of neuroticism are associated with altered VAN connectivity (Lee et al., 2024; Simon et al., 2020; Wang and Chen, 2024) that that has been linked to altered attentional processing and potentially emotion dysregulation; this alteration may mediate the relationship between neuroticism and emotional disturbances, such as depressive symptoms (Lee et al., 2024). Furthermore, increased VAN-DMN connectivity has been highlighted as a mediating mechanism explaining how neuroticism contributes to general appearance dissatisfaction, potentially reflecting an abnormal engagement of VAN-mediated attentional processes at rest, leading to excessive self-focus on body image concerns (Wang and Chen, 2024). These findings underscore the VAN as a critical brain network in understanding vulnerability to neuroticism and highlight its potential as a target for therapeutic interventions. The consistency of our findings across different sphere sizes (1, 4, and 7 mm) underscores the robustness and reliability of the identified dysfunctional networks linked to neuroticism. This consistency suggests that the observed findings are not merely due to methodological artifacts or specific parameter choices, but rather reflect a genuine and replicable association between these brain networks and neuroticism.

Furthermore, our primary findings revealed that the spatial distribution of these identified networks was significantly correlated with the atlas-based distributions of the 5-HT2A, CB1, and mGluR5. This novel cross-modal association between brain networks and neurotransmitter atlases strongly aligns with and is robustly supported by a wealth of direct PET imaging studies, collectively highlighting a critical role for the 5-HT2A receptor in the neurobiology of neuroticism. For example, seminal work by Frokjaer et al. (2008) and recent replications by Høgsted et al. (2024) consistently found positive correlations between 5-HT2A receptor binding in fronto-limbic and neocortical regions, respectively, and neuroticism scores, particularly its introspective facets. Underscoring its clinical relevance, Sankar et al. (2024) demonstrated that high neocortical 5-HT2A binding, interacting with introspective neuroticism, predicts future depression risk. This neurobiological link is further, albeit indirectly, supported by genetic studies on HTR2A polymorphisms (Fiocco et al., 2007; Golimbet et al., 2002), which, despite complexities and potential influences like clinical status or sex differences in binding (Soloff et al., 2010), point to the HTR2A gene’s involvement in neuroticism. Mechanistically, it is highly probable that the neuroticism-associated networks we identified overlap significantly with these 5-HT2A-rich brain regions—such as the PFC, anterior cingulate cortex, insula, and limbic areas (Frokjaer et al., 2008; Høgsted et al., 2024; Soloff et al., 2010), which are critical for emotional processing and stress response. Our investigation additionally identified a significant positive spatial correlation between neuroticism-associated networks and the distribution of CB1, pointing to the endocannabinoid system’s (ECS) involvement in the neuroanatomical substrate of this personality trait. The spatial coupling in our study via JuSpace toolbox analysis is consistent with several genetic studies (Aleksandrova et al., 2012; Juhasz et al., 2009) and neurochemical research (Kolla et al., 2022), and the broader understanding of the ECS’s role in emotion and stress (Navarrete et al., 2020). Soundararajan et al. (2021) provided valuable contemporary genetic evidence that supports the plausibility of CB1’s involvement in neuroticism, particularly by showing how a CB1 SNP can influence sleep disturbances through neuroticism in a non-clinical population. While the association between single genes and complex traits is challenging and influenced by multiple factors, our finding, from a neuroanatomical perspective, reinforces the importance of CB1 in the neurobiological mechanisms of neuroticism. This could offer new targets for interventions aimed at neuroticism-related traits and their associated emotional consequences (Navarrete et al., 2020). Our finding of a significant positive spatial correlation between neuroticism-associated networks and mGluR5 receptor distribution gains substantial support from multiple lines of evidence, positioning the glutamatergic system, and mGluR5 specifically, as a key player in the neurobiology of neuroticism. Hasler et al. (2019) reported that higher prefrontal glutamine levels—a marker indicative of increased glutamatergic activity—were positively correlated with neuroticism in young adults, and they hypothesized that mGluR5 might be downregulated in response to such activity. This human *in vivo* neurochemical link is powerfully complemented by the recent Mendelian randomization study by Narayan et al. (2024), which identified GRM5, the gene encoding mGluR5, as one of six top candidate genes for Alzheimer’s disease (AD) therapeutics, with neuroticism causally mediating the pathway from loneliness/social isolation to AD risk (Narayan et al., 2024). This convergence suggests mGluR5 is not only linked to the trait of neuroticism itself but also to its potential downstream pathological consequences.

It is noteworthy that while our study, utilizing validated neurotransmitter atlases via the JuSpace toolbox, reveals significant spatial coupling between neuroticism-associated brain networks and mGluR5 and CB1 receptor distributions, direct *in vivo* PET imaging studies investigating the availability of these specific receptors in relation to neuroticism are considerably less extensive compared to those for the 5-HT2A receptor. PET research on the 5-HT2A receptor has a longer history and broader application, with more mature and diverse radiotracers available. In contrast, the development and application of specific PET tracers for mGluR5 and CB1 in the context of personality traits are still evolving, which has, to some extent, limited direct *in vivo* validation of their roles in neuroticism. Therefore, our findings not only underscore the potential importance of the glutamatergic and endocannabinoid systems in the neurobiology of neuroticism but also provide a clear directive for future research. Future investigations employing specific mGluR5 and CB1 PET radiotracers in cohorts well-characterized for neuroticism are crucial. Such studies would allow for: (1) direct validation of whether the spatial associations observed at the atlas level in our study reflect actual differences in receptor density or availability; (2) deeper mechanistic insights into the specific roles these receptor systems play in the functional and structural brain alterations associated with neuroticism; and (3) more direct biological evidence for the development of novel therapeutic targets for neuroticism-related emotional disorders, such as anxiety and depression. This represents a critical next step in filling the current knowledge gap and advancing our understanding of the neurobiology of neuroticism from a molecular imaging perspective.

## Limitations

Several limitations should be considered when interpreting our findings. First, we acknowledge a demographic mismatch between the diverse populations in the original VBM studies and the healthy young adults in the HCP normative connectome. While age-related variations in FC may create bias, the macroscale network architecture is generally deemed stable enough to produce consistent localizations throughout adulthood. To enhance the accuracy of these network maps, future research should incorporate age-stratified or population-specific connectomes. Second, while the FCNM approach provides a valuable network-level perspective, it is constrained by the spatial resolution and methodological limitations of VBM and fMRI techniques. Subtle changes or more complex network interactions may not be fully captured by this approach. Third, the current FCNM approach focused on healthy individuals. Future research should investigate whether these network-level GM alterations are also present in clinical populations characterized by high neuroticism, such as individuals with anxiety disorders or depression, to assess the clinical relevance of these findings. Fourth, our analysis was limited to a non-clinical population. Since high neuroticism is a significant vulnerability factor for clinical disorders like major depressive disorder and anxiety, the next crucial step is to broaden this study to include patient populations. Future studies should examine whether the identified tripartite network (DMN, FPN, VAN) is similarly dysregulated in these clinical groups and critically, whether such network alterations mediate the pathway from neuroticism to clinical symptom severity. Fifth, while our findings contribute to understanding functional connectome-neuroticism mapping, they must be interpreted in light of persistent methodological challenges in neuroimaging research, including factors known to impact reproducibility such as small sample sizes, sample heterogeneity, experimental variability, and analytical flexibility. Advancing this field crucially depends on continued dedication to standardization, recruitment of larger and more diverse cohorts, and transparent reporting. Notwithstanding these constraints, FCNM is a rapidly evolving discipline with growing interest, poised for significant advancements through continued technological innovation. Finally, the JuSpace analysis relies on spatial correlations between MRI-derived network maps and average neurotransmitter atlas data. This does not directly measure receptor density or function in the individuals whose data contributed to the neuroticism networks. Future research should aim to validate these findings using PET imaging or pharmacological challenge studies to test receptor-level differences, specifically focusing on 5-HT2A, CB1, and mGluR5 availability and function in individuals with high and low neuroticism within the identified network nodes.

## Conclusion

This study, for the first time, integrated the FCNM approach with large-scale human brain connectome data from the HCP to reveal that heterogenous GM correlates of neuroticism commonly mapped onto specific brain networks. Our findings indicated that aberrant brain networks linked to neuroticism predominantly implicate the DMN, FPN, and VAN. These disrupted brain networks may be associated with core features of neuroticism, including heightened self-referential processing, impaired cognitive control, and increased sensitivity to salient stimuli. Network localization offers a comprehensive and unified framework that may help address concerns regarding the reproducibility of GM findings across VBM studies in personality neuroscience. Furthermore, this study provides novel evidence suggesting that the brain network associated with GM correlates of neuroticism is characterized by a specific neurochemical signature involving 5-HT2A, CB1, and mGluR5 receptors. These findings bridge the gap between macroscale brain networks and microscale neurotransmitter systems. By highlighting the roles of serotonergic, endocannabinoid, and glutamatergic modulation within a trait-relevant network, our results may offer new avenues for research into the biological basis of neuroticism and potential targets for interventions aimed at mitigating its impact on mental health.

## Data Availability

The raw data supporting the conclusions of this article will be made available by the authors, without undue reservation.

## Footnotes

1
https://www.sdmproject.com/utilities/?show~=~Coordinates

2
https://www.fil.ion.ucl.ac.uk/spm/

3
https://rfmri.org/DPABI

4
https://github.com/juryxy/JuSpace

## Supplementary material

Supplementary Figure 1 Flowchart of literature search and study selection process. GM, gray matter; VBM, voxel-based morphometry; ROI, region of interest.

Supplementary Figure 2 Neuroticism-associated FC overlap map (1-mm sphere radius). Functional brain networks are shown as FC probability maps thresholded at 50%, showing brain regions functionally connected to more than 50% of the contrast seeds. FC, functional connectivity; L, left; R, right.

Supplementary Figure 3 Neuroticism-associated FC overlap map (7-mm sphere radius). Functional brain networks are shown as FC probability maps thresholded at 50%, showing brain regions functionally connected to more than 50% of the contrast seeds. FC, functional connectivity; L, left; R, right.

Supplementary Figure 4 Neuroticism-associated FC overlap maps based on 1-mm radius sphere in association with canonical brain networks. Polar plots illustrate the proportion of overlapping voxels between each neuroticism FC map and a canonical network to all voxels within the corresponding canonical network. The blue dot represents brain functional networks, defined as significant networks, exhibiting ≥ 10% overlap with canonical networks, whereas the orange dot represents non-significant networks with < 10% overlap. FC, functional connectivity.

Supplementary Figure 5 Neuroticism-associated FC overlap maps based on 7-mm radius sphere in association with canonical brain networks. Polar plots illustrate the proportion of overlapping voxels between each neuroticism FC map and a canonical network to all voxels within the corresponding canonical network. Note: The blue dot represents brain functional networks, defined as significant networks, exhibiting ≥ 10% overlap with canonical networks, whereas the orange dot represents non-significant networks with < 10% overlap. FC, functional connectivity.
