# Supplementary material for: Network-based mapping and neurotransmitter architecture of gray matter correlates of neuroticism
Source: Front Syst Neurosci. 2026 Jan 8;19:1713434. doi: 10.3389/fnsys.2025.1713434 (PMC12823854; doi:10.3389/fnsys.2025.1713434)
Supplement: Supplementary file 1 [file Table_1.docx]

**Table S1.** Demographic information of the HCP

| **Dataset sample size** | **Age (years)** | **Gender (F/M)** |
| --- | --- | --- |
| HCP 1093 | 28.78 ± 3.69 | 594/499 |

Age is expressed as mean ± standard deviation. Note: HCP, Human Connectome Project; F, female;

M, male.
