# Supplementary figures and images for: Network-based mapping and neurotransmitter architecture of gray matter correlates of neuroticism
Source: Front Syst Neurosci. 2026 Jan 8;19:1713434. doi: 10.3389/fnsys.2025.1713434 (PMC12823854; doi:10.3389/fnsys.2025.1713434)

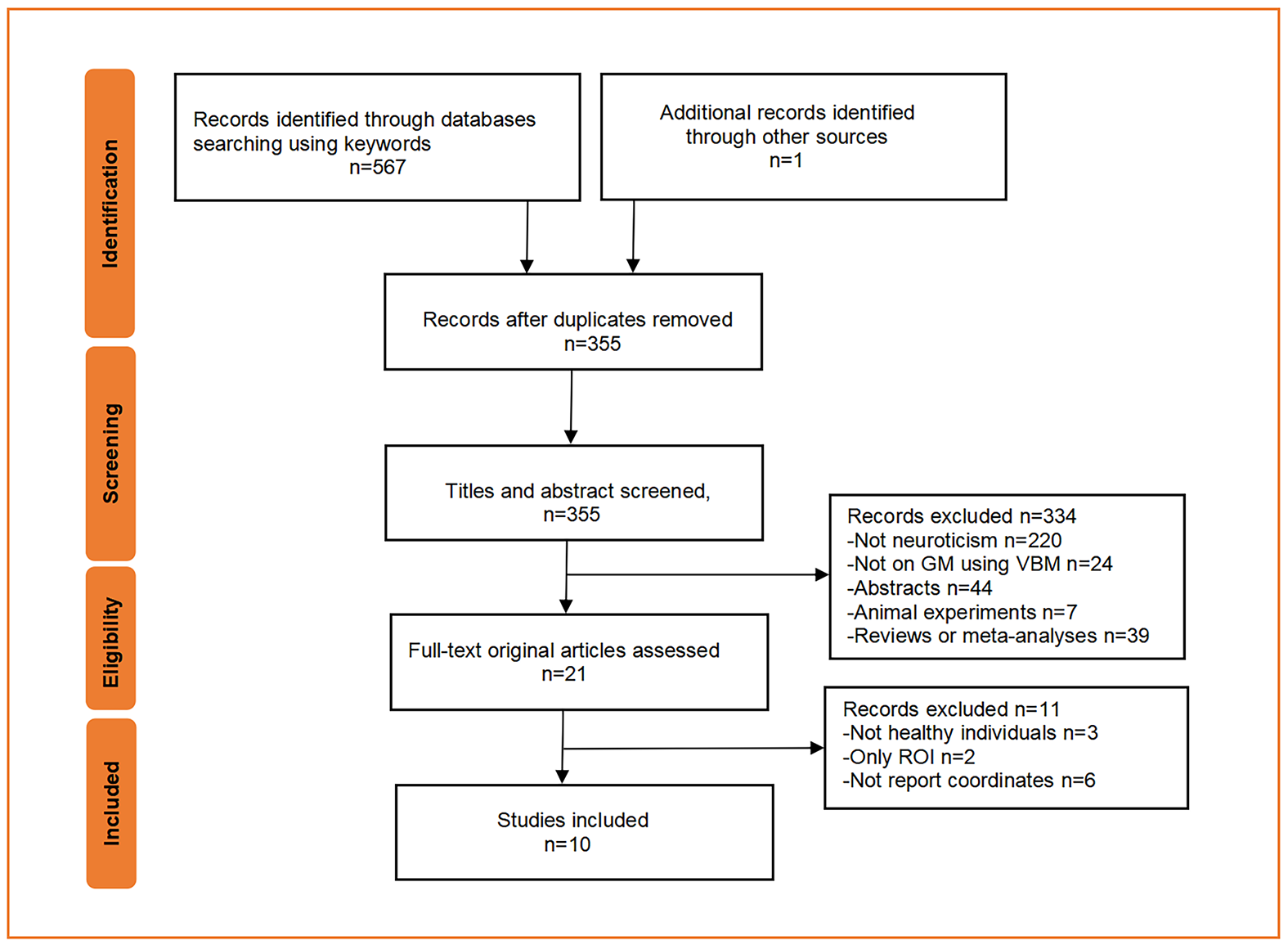

Supplement: Supplementary Figure 1 — Flowchart of literature search and study selection process. GM, gray matter; VBM, voxel-based morphometry; ROI, region of interest. [file Image_1.tif]

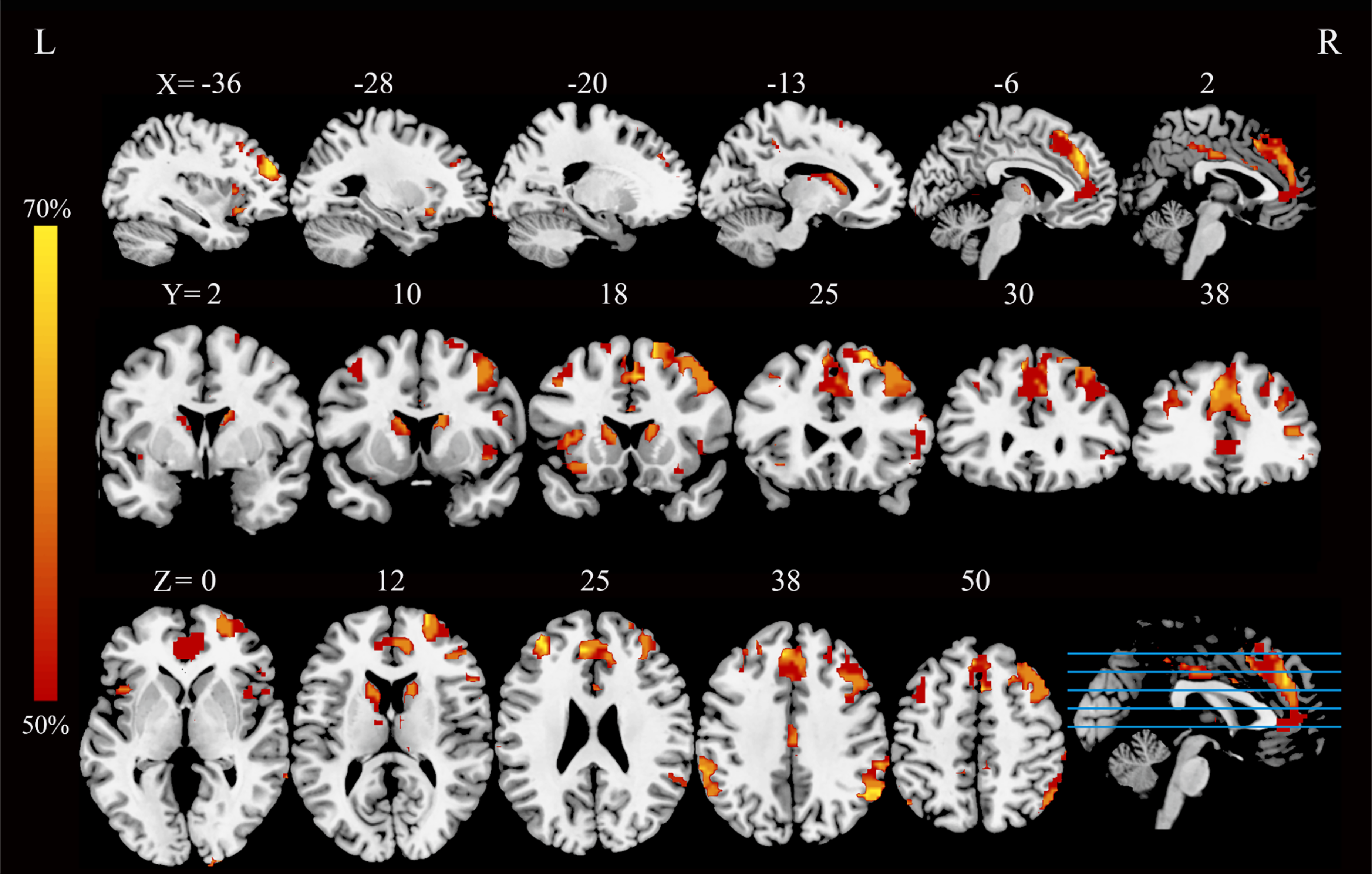

Supplement: Supplementary Figure 2 — Neuroticism-associated FC overlap map (1-mm sphere radius). Functional brain networks are shown as FC probability maps thresholded at 50%, showing brain regions functionally connected to more than 50% of the contrast seeds. FC, functional connectivity; L, left; R, right. [file Image_2.tif]

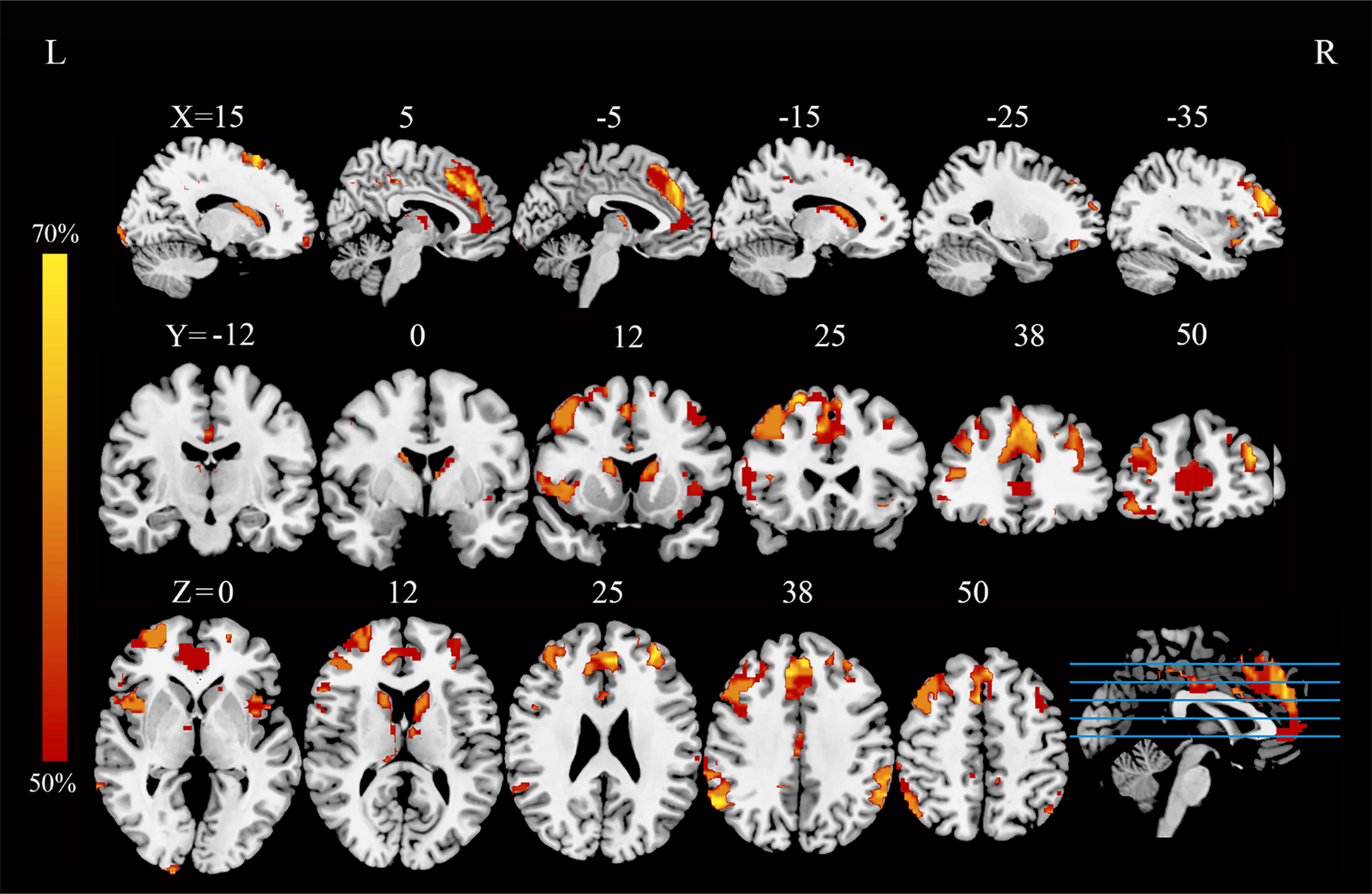

Supplement: Supplementary Figure 3 — Neuroticism-associated FC overlap map (7-mm sphere radius). Functional brain networks are shown as FC probability maps thresholded at 50%, showing brain regions functionally connected to more than 50% of the contrast seeds. FC, functional connectivity; L, left; R, right. [file Image_3.tif]

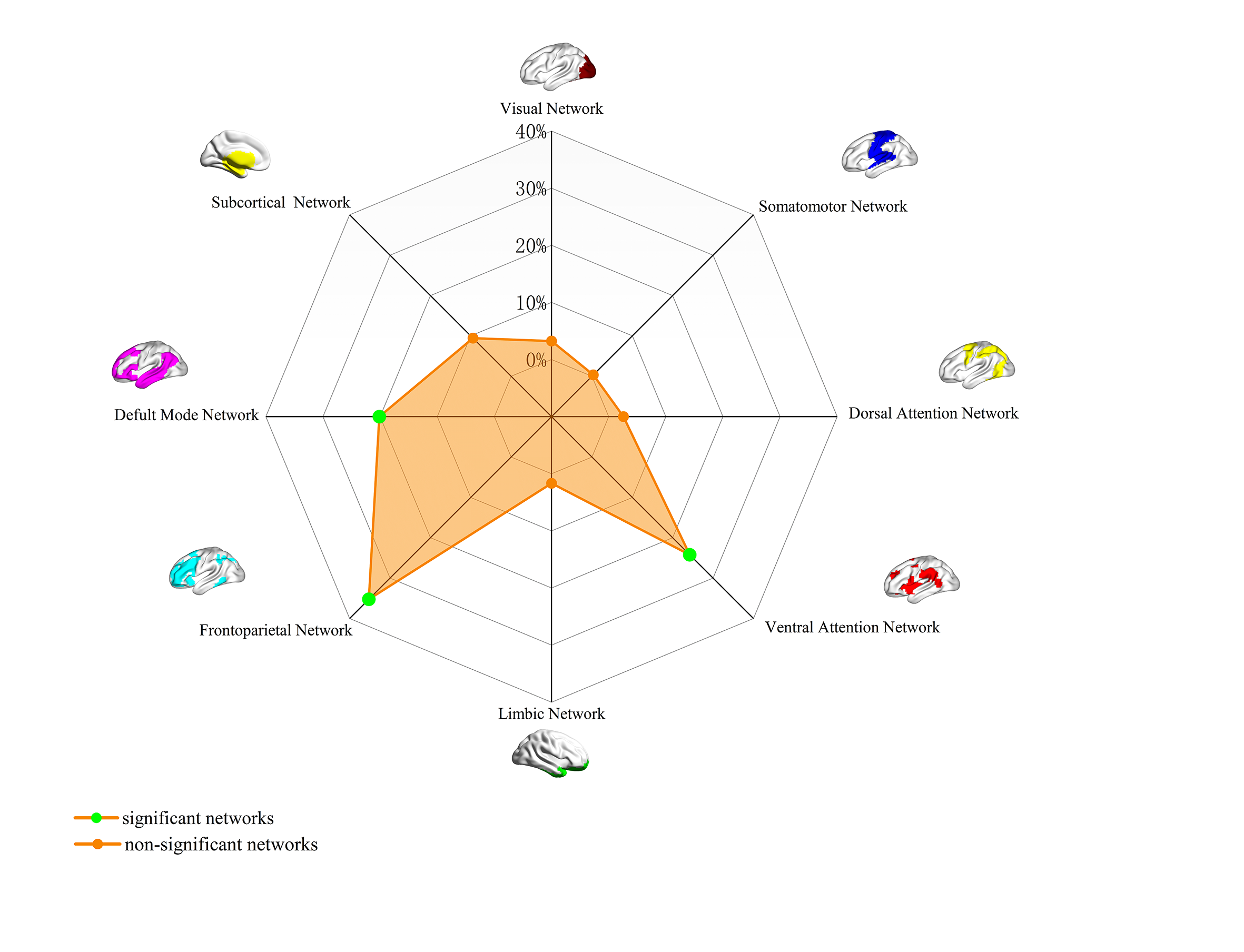

Supplement: Supplementary Figure 4 — Neuroticism-associated FC overlap maps based on 1-mm radius sphere in association with canonical brain networks. Polar plots illustrate the proportion of overlapping voxels between each neuroticism FC map and a canonical network to all voxels within the corresponding canonical network. The blue dot represents brain functional networks, defined as significant networks, exhibiting ≥ 10% overlap with canonical networks, whereas the orange dot represents non-significant networks with < 10% overlap. FC, functional connectivity. [file Image_4.tif]

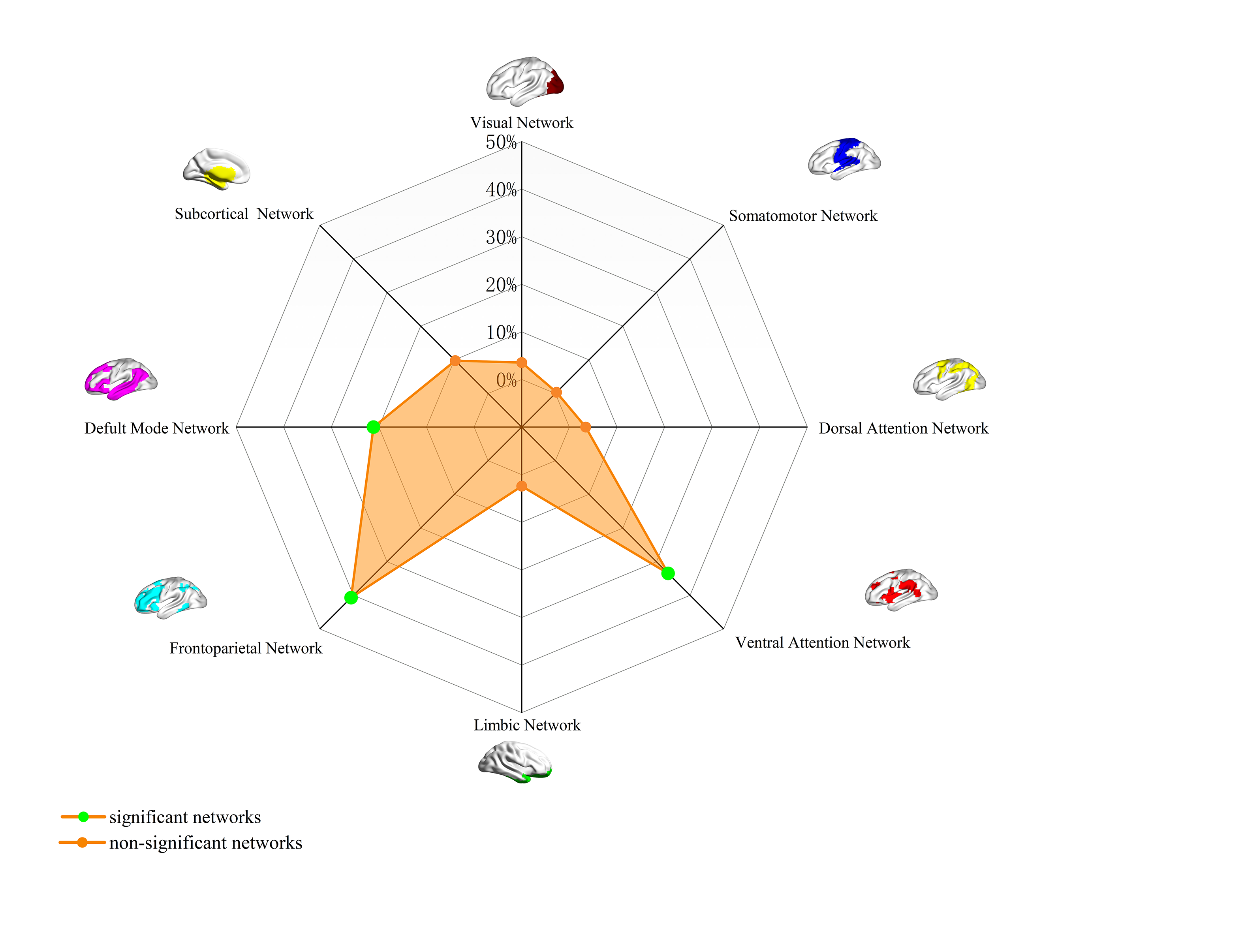

Supplement: Supplementary Figure 5 — Neuroticism-associated FC overlap maps based on 7-mm radius sphere in association with canonical brain networks. Polar plots illustrate the proportion of overlapping voxels between each neuroticism FC map and a canonical network to all voxels within the corresponding canonical network. Note: The blue dot represents brain functional networks, defined as significant networks, exhibiting ≥ 10% overlap with canonical networks, whereas the orange dot represents non-significant networks with < 10% overlap. FC, functional connectivity. [file Image_5.tif]
